# Addressing mental health need after COVID-19: a systematic review of remote EMDR therapy studies as an emerging option

**DOI:** 10.3389/fpsyt.2023.1336569

**Published:** 2024-01-04

**Authors:** Safa Kemal Kaptan, Zehra Merve Kaya, Ayşe Akan

**Affiliations:** ^1^Assistant Professor, Department of Psychology, Boğaziçi University, İstanbul, Türkiye; ^2^Honorary Fellow of the Institute of Teaching and Learning at the University of Manchester, Manchester, United Kingdom; ^3^Visiting Scholar, Department of Psychology, Boğaziçi University, İstanbul, Türkiye; ^4^Licensed Clinical Psychologist, Chicago, State of Illinois, IL, United States; ^5^Registered Clinical Psychologist, Health and Care Professions Council (HCPC), London, United Kingdom

**Keywords:** EMDR therapy, online therapy, mental health, online EMDR, remote EMDR, self-help

## Abstract

**Introduction:**

The COVID-19 pandemic has been associated with a substantial rise in mental health challenges, prompting a need for accessible and effective therapeutic interventions. This review summarizes the evidence on remote Eye Movement Desensitization and Reprocessing (EMDR) therapy delivered in response to the increased need.

**Methods:**

A systematic review was conducted following the Preferred Reporting Items for Systematic Reviews and Meta-Analyses (PRISMA) guidelines. Databases including PsychINFO, EMBASE, MEDLINE, and Web of Science were searched to identify studies assessing the efficacy of EMDR therapy administered online.

**Results:**

Sixteen articles meeting the inclusion criteria were selected, involving 1,231 participants across various age groups. Studies covered remote individual and group EMDR sessions and self-administered computerized protocols. Findings indicate promising outcomes in reducing PTSD symptoms, anxiety, and depression.

**Discussion:**

The analysis of the selected studies demonstrates the feasibility and potential efficacy of online EMDR as an accessible therapeutic option for addressing mental health difficulties, particularly during times of limited in-person interaction. However, the studies revealed limitations such as small sample sizes, absence of control groups, and reliance on self-reported measures.

**Systematic review registration:** The present review was registered on “The International Database to Register Your Systematic Reviews” (INPLASY) with the registration number 2023120018 and DOI number 10.37766/inplasy2023.2.0068.

## 1 Introduction

The COVID-19 pandemic has been associated with a significant increase in mental health difficulties, including post-traumatic stress, anxiety, and depression (1). Studies have indicated that individuals reporting symptoms of anxiety or depression were more prone to severe COVID-19 outcomes (2). Further research has shown that individuals who contracted COVID-19 also experienced elevated levels of anxiety, depression, post-traumatic symptoms, and other difficulties (3, 4).

The pandemic affected mental health through different factors such as isolation, financial instability, grief, suicide, and substance use (5). Different psychological impacts persisted, leading to fatigue, cognitive impairments, and ongoing mental health difficulties even in many post-infection cases (6). Specific groups, including racial and ethnic minorities, displaced groups, individuals with financial insecurity, children, people with disabilities, and those with preexisting conditions, faced an even more heightened risk (7). Essential workers, for example, experienced worsened mental health due to the increased risk of contracting or becoming severely ill from COVID-19 (3).

The global increase in mental health difficulties during the pandemic was met with limited access to mental health services and resources due to restrictions such as lockdowns or the prioritization of other health services. The disruptions caused by COVID-19 led to a decrease in access to outpatient mental health care, reduced admissions, and earlier discharge from inpatient care (8). In European countries where rates of depression and anxiety among the general population are high, long waiting lists have been identified as a barrier to mental health services (9, 10).

In response to these challenges, there was a shift towards providing remote services and using technology in mental health care. Digital tools have supported mental health services for over two decades. However, the COVID-19 pandemic created a unique need for greater utilization of these digital technologies to offer effective and timely solutions that scale up and decentralize health care across a wide variety of platforms, including teletherapy, mHealth (mobile health) applications, and web-based interventions or self-help tools (11–13).

Eye Movement Desensitization and Reprocessing (EMDR) is a psychotherapy designed to alleviate distress associated with traumatic memories. It involves attention to three time periods: the past, present, and future, with a focus on past disturbing memories and related events (14). EMDR therapy is an eight-phase treatment that uses eye movements (or other bilateral stimulation) during one part of the session (15, 16). Research indicates that EMDR therapy can be an effective treatment for difficulties such as anxiety, depression, chronic pain, addictions, and other distressing life experiences (17–23).

With the emergence of the COVID-19 pandemic, like other forms of therapy, online EMDR therapy has gained widespread popularity. Following the widespread application, many EMDR organizations such as EMDR Europe, EMDR Association UK, and EMDR International Association (EMDRIA) have issued guidelines offering specific recommendations regarding security, therapeutic considerations, and client selection in this context (24). Qualitative studies have also shed light on the feasibility and accessibility of online EMDR therapy during the pandemic. For example, in a study conducted in the UK with therapists, most participants expressed high comfort in receiving EMDR therapy online (25). It suggested many therapists who were initially hesitant about doing EMDR therapy online decreased considerably within the first year of the pandemic. Moreover, four-fifths of therapists intended to continue offering online therapy even after pandemic restrictions were lifted. The number of therapists not delivering any EMDR therapy sessions online decreased significantly, and this trend continued to decline thereafter. In another study (26), some clinicians initially expressed questions about engaging in EMDR therapy, mainly due to the physicality involved, such as the bilateral stimulation. Despite their initial hesitation, clinicians recognized the value of integrating EMDR into their therapeutic toolkit and highlighted the significance of employing such approaches when working with clients under different circumstances.

The effectiveness of online Eye Movement Desensitization and Reprocessing (EMDR) is not well-established, with previous systematic reviews and meta-analyses concentrating on face-to-face applications. A notable gap exists in the literature regarding a systematic review summarizing the evidence for online EMDR. This review aims to fill this gap by focusing on remote EMDR interventions, contributing valuable insights to the existing literature (See Table 1).

**Table 1:** Study characteristics.

Authors	N in EMDR	Sample group	Study design	Comparison group	Type of protocol	Facilitator	Delivery format	Number of sessions	Longest follow up
Bates et al., 2022	13	Adults Mean age not specified	RCT	Usual care	R-TEP	EMDR therapists	Individual live sessions	Up to 8	6 months
Farrell et al., 2023	95	Frontline healthcare workers Mean age not specified	RCT	A delayed treatment	G-TEP	EMDR therapists	Group live sessions (6 participants)	4 sessions	6 months
Moench et al., 2021	34	Healthcare workers Mean age not specified	RCT	A delayed treatment	STEP	NA	Self-administered computerized sessions	Single session	NA
Clarke, 2022	1	Adults Mean age not specified	Single arm	Pre-post	R-TEP	EMDR therapists	Individual live sessions	9 sessions	4 months
Faretta et al., 2022	11	Frontline healthcare workers 7 females 4 males Mean age not specified	Single arm	Pre-post	IGTP	EMDR therapists	Group live sessions	3 sessions	9 months
Farrell et al., 2022	24	Frontline healthcare workers Mean age not specified	Single arm	Pre-post	Blind 2 Therapist	EMDR therapists	Individual live sessions	Single session	6 months
Fernandez et al., 2022	587	Frontline healthcare workers 433 females 154 males, Mean age 45.5	Single arm	Pre-post	IGTP	EMDR therapists	Group live sessions (4–6 participants)	3 sessions	NA
Goga et al., 2022	31	Adults 14 males 17 females Mean age 26.2	Single arm	Pre-post	Standard EMDR	NA	Self-administered computerized sessions	Single session	NA
Lazzaroni et al., 2021	50	Adolescents and young adults Aged 13 to 24 years Mean age not specified	Single arm	Pre-post	R-TEP	EMDR therapists	Group live sessions (6 participants)	3 sessions	NA
McGowan et al., 2021	93	Adolescents and Adults 10 to 72 years Mean age 35.5	Single arm	Pre-post	Standard EMDR	EMDR therapists with an average experience of 8.5 years	Individual live sessions	Unclear	NA
Mischler et al., 2021	76	Adults 18 to 68 years Mean age 41	Single arm	Pre-post	Standard EMDR	EMDR therapists with an average experience of 10.7 years	Individual live sessions	On average, patients had received 4.84 eEMDR sessions (SD = 5.28, range = 0–30)	NA
Morris et al., 2022	12	Healthcare workers Mean age not specified	Single arm	Pre-post	R-TEP	EMDR therapists	Individual live sessions		
Perri et al., 2021	19	Adults males Mean age 48.3	Single arm	Pre-post	R-TEP	EMDR therapists	Individual live sessions	7 sessions	1 month
Sagaltici et al., 2022	14	Healthcare workers 3 males 11 females Mean age not specified	Single arm	Pre-post	R-TEP	EMDR therapists	Individual live sessions	5 sessions	1 month
Tarquinio et al., 2020	17	Healthcare workers 17 females Mean age 33.2	Single arm	Pre-post	URG-EMDR protocol	EMDR therapists	Individual live sessions	Single session	NA
Yurtsever et al., 2022	154	Frontline workers Mean age not specified	Single arm	Pre-post	R-TEP	EMDR therapists	Individual live sessions	5 sessions	1 month

## 2 Methods

### 2.1 Design

The protocol of the present systematic review was registered retrospectively on “The International Database to Register Your Systematic Reviews” (INPLASY) with the registration number 2023120018 and DOI number 10.37766/inplasy2023.12.0018. The study was guided by the Preferred Reporting Items for Systematic Reviews and Meta-Analyses (PRISMA) (27). The purpose of this review was to comprehensively summarize the effectiveness of EMDR therapy based on existing literature. The inclusion criteria encompassed studies that assessed the efficacy of any EMDR, provided that EMDR sessions were delivered online and the study employed at least one standardized outcome that measures mental health difficulties. Trials with or without a control group were both considered. The inclusion criteria were not restricted based on publication date, study design, setting, age, gender, or publication status, except for requiring the studies to be in English. However, studies that did not present primary quantitative findings on the effectiveness of EMDR in peer-reviewed publications, such as reviews, books, conference abstracts, or posters, were excluded. Additionally, studies that combined the EMDR with other therapies or interventions without providing appropriate statistical differentiation of EMDR effects were also excluded.

### 2.2 Search

A database search was conducted across multiple databases, including PsychINFO, EMBASE, MEDLINE, and Web of Science, between 1989 and September 2023. Additionally, the Francine Shapiro Library and the Journal of EMDR Practice and Research were hand-searched renowned for focusing on EMDR studies. To ensure inclusivity, ongoing studies were searched from the UK Clinical Trials Gateway and The ISRCTN Registry. The searches were repeated to guarantee the most current evidence for synthesis before finalizing the review. The search terms employed in all databases included combinations of terms *Eye Movement Desensitization and Reprocessing (EMDR)*, along with *remote, online*, and *web based*. Furthermore, the reference lists of eligible studies as well as previous systematic reviews and meta-analyses pertaining to EMDR were searched for further studies. Two independent reviewers conducted the study selection process in two phases – initially focusing on titles and abstracts, followed by a comprehensive evaluation of full-text articles. Disagreements were solved through discussion or arbitration with a third reviewer. A data extraction sheet was developed, initially pilot-tested and subsequently refined. The data extraction covered various elements such as detailed sample and study design information. Additionally, specifics about the EMDR protocol were all extracted from each study.

### 2.3 Quality assessment

The risk of bias was evaluated across articles by using A Revised Tool to Assess the Risk of Bias in Randomised Trials (RoB-2) (28) and the Risk of Bias in Non-randomized Studies of Interventions (ROBINS-1) (29).

### 2.4 Analysis

Given the significant heterogeneity in terms of study design, interventions, outcomes, follow-up periods, and sample characteristics, a meta-analysis was not feasible. Consequently, the study opted for a narrative synthesis approach to interpret and present the findings from the diverse studies included in this review. This method provided a descriptive and comprehensive account of the results, highlighting trends, patterns, and variations within the available evidence.

## 3 Findings

### 3.1 Study characteristics

**Figure 1:**
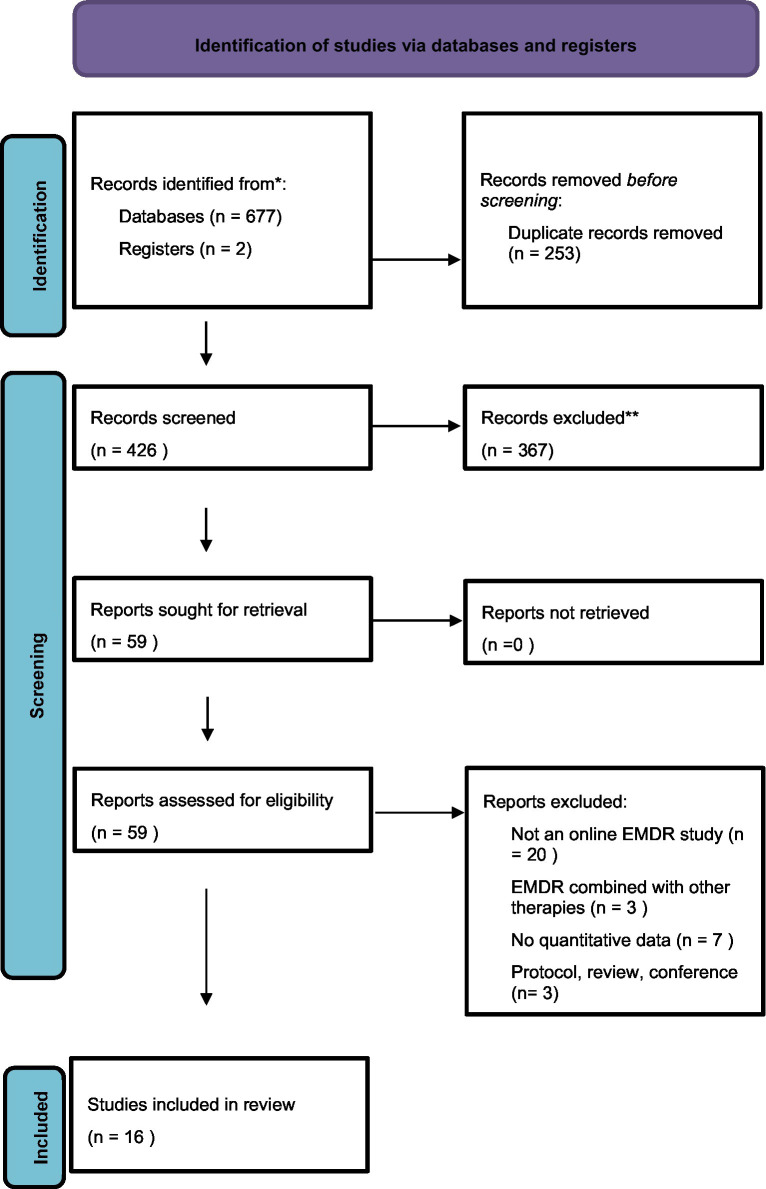
Flowchart of the systematic review process.

The flow of studies is displayed in Figure 1. Sixteen articles were included in the review.

Participants: the total participant count across the 16 eligible studies was 1,231 individuals. The sample sizes showed considerable variation and encompassed both adolescents and adults. Among the studies, 14 recruited adult participants exclusively, while two had mixed groups comprising adolescents and adults. Notably, nine studies specifically focused on healthcare workers within their samples. Different studies utilize individual live sessions or group live sessions, showing flexibility in therapeutic delivery formats. Self-administered computerized sessions are also used in some cases.

Interventions: the treatment durations ranged from a single session to nine sessions, with each session ranging between 60 min and 134 min. The studies tested seven different protocols (R-TEP, G-TEP, IGTP, Standard EMDR protocol, STEP, Blind 2 Therapist, and URG-EMDR protocol), indicating the adaptability of EMDR to address various forms of trauma or distress. Notably, seven articles tested the efficacy of The Recent Traumatic Episode (R-TEP) protocol while 3 articles tested standard EMDR protocol.

Comparator/Design: studies incorporated randomised controlled trials and single-arm pre-post designs. Yet the majority of the studies used a one-arm design with pre- and post-treatment assessments. The follow-up durations ranged from 1 month to 9 months.

Outcomes: across all studies, the central focus was on the alteration of PTSD scores as the primary outcome. Secondary outcomes encompassed depression, anxiety, and Subjective Units of Disturbance (SUD), which is a patient-report scale that measures the distress the client feels about the particular memory that is being processed during the EMDR session. Studies used various tools such as PTSD CheckList – Civilian Version (PCL-C), PTSD Checklist for DSM-5 (PCL-5), Post-traumatic Stress Symptoms-14 (PTSS-14), The International Trauma Consortium (ITQ), Impact of Event Scale – Revised (IES-R), Depression, Stress and Anxiety Scale (DASS 21) and Hospital Anxiety and Depression Scale (HADS) to assess symptoms related to PTSD, stress, and trauma. Patient Health Questionnaire-9 (PHQ-9), Beck’s Depression Inventory (BDI-II), HADS, and DASS-21 were utilized across different studies to evaluate depressive symptoms. Generalised Anxiety Disorder Assessment (GAD-7), The State-Trait Anxiety Inventory (STAI), BDI-II, and HADS were used to measure symptoms of anxiety. Several studies evaluate SUD using different assessment tools. Additional measures, including burnout assessment tools like The Brief Resilience Scale (BRS), Maslach Burnout Inventory (MBI), and instruments for self-efficacy (General Self-Efficacy Scale [GSE]), post-traumatic growth (post traumatic growth inventory [PTGI]), and Mental Illness-Related Shame (MIES) were utilized in some studies. For outcomes, please refer to Table 2.

**Table 2:** Outcomes.

	PTSD/Stress	Depression	Anxiety	SUD	Others
Bates et al., 2022	PCL-C+	HADS	HADS		EQ-5D-5L BRS
Farrell et al., 2023	ITQ+	PHQ-9+	GAD-7+	SUD+	MIES
Moench et al., 2021		DASS-21+	DASS-21+		GSE+
Clarke, 2022	PTSS-14+	PHQ-9+	GAD-7+		EQ-5D-5L+
Faretta et al., 2022	IES-R				THERMO+
Farrell et al., 2022				SUD+	
Fernandez et al., 2022	IES-R+				THERMO+
Goga et al., 2022	IES-R+		STAI+	SUD+	
Lazzaroni et al., 2021	IES-R+		STAI+		THERMO+ PTGI
McGowan et al., 2021	IES(R)+ PCL-5+	PHQ-9+	GAD-7+		
Mischler et al., 2021				SUD+	
Morris et al., 2022	PCL-5+			SUD+	
Perri et al., 2021	PCL-5+	BDI-II+	STAI+		
Sagaltici et al., 2022	IES-R+	BDI-II+	BAI+		MBI+
Tarquinio et al., 2020		HAD+	HAD+	SUD+	
Yurtsever et al., 2022	IES-R+				

+Significant improvement at the final measurement point from the baseline or control group. PTGI, post-traumatic growth inventory; BRS, the brief resilience scale; MBI, Maslach burnout inventory; IES-R, the impact of event scale-revised; PCL-5, the PTSD checklist for DSM-5; HADS, the hospital anxiety and depression scale; BDI-II, the beck depression inventory-II; STAI, the state-trait anxiety inventory; SUD, subjective units of disturbance scale; PTGI, the post-traumatic growth inventory; PCL-C, the post-traumatic stress disorder checklist-Civilian; BAI, beck anxiety inventory; PTSS-14, the post-traumatic stress syndrome 14-questions inventory; The EQ-5D, EuroQol Group; SDS, the sheehan disability scale; DASS-21, depression and anxiety stress scale; GSE, the generalized self-efficacy scale; PCL-C, PTSD checklist; PSS-I, the PTSD symptom scale-interview; THERMO, the emotion thermometer; MIES, moral injury event scale; K10, the Kessler 10 item.

### 3.2 Quality assessment

**Figure 2:**
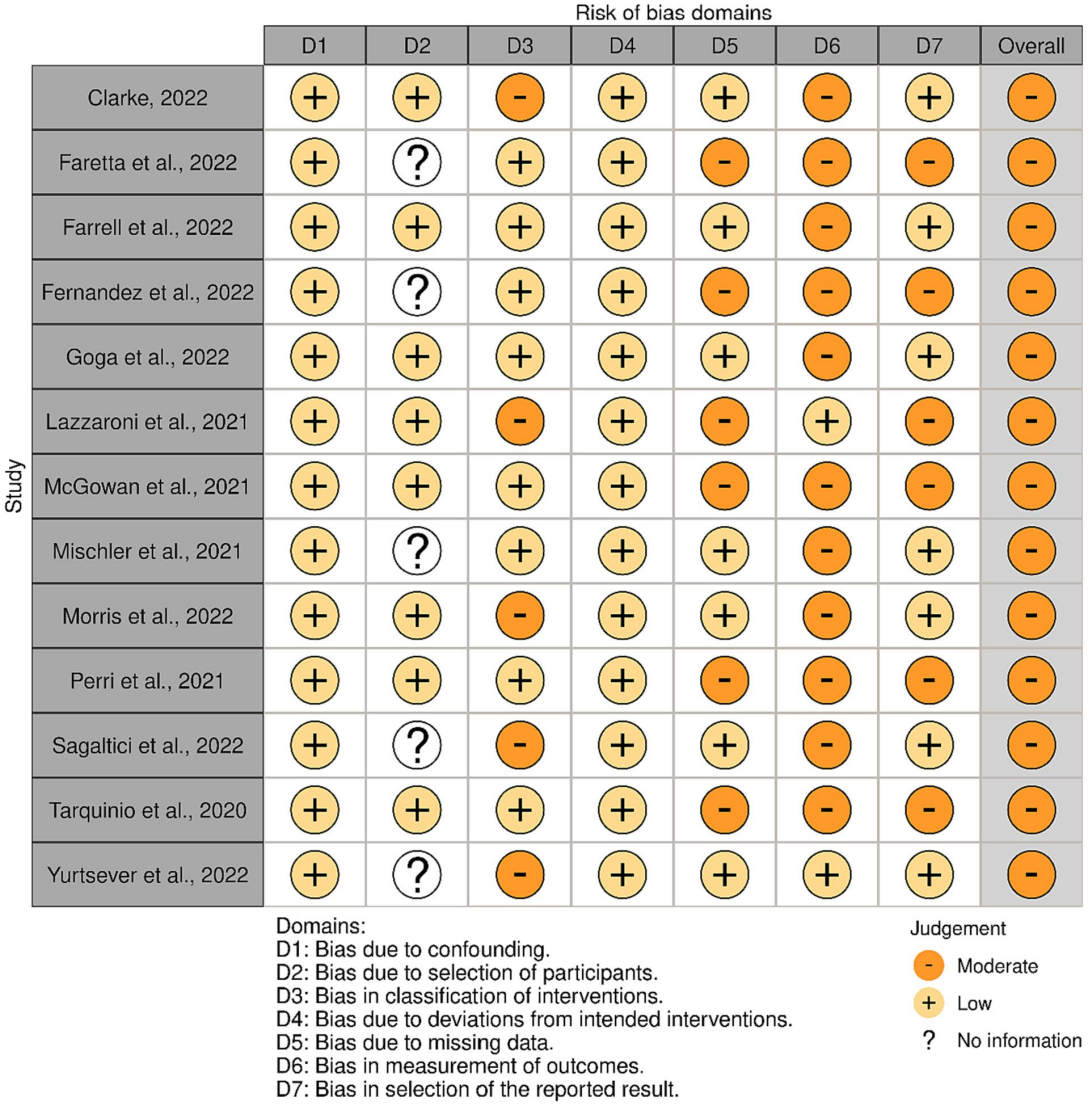
Quality evaluation of non-randomised studies.

**Figure 3:**
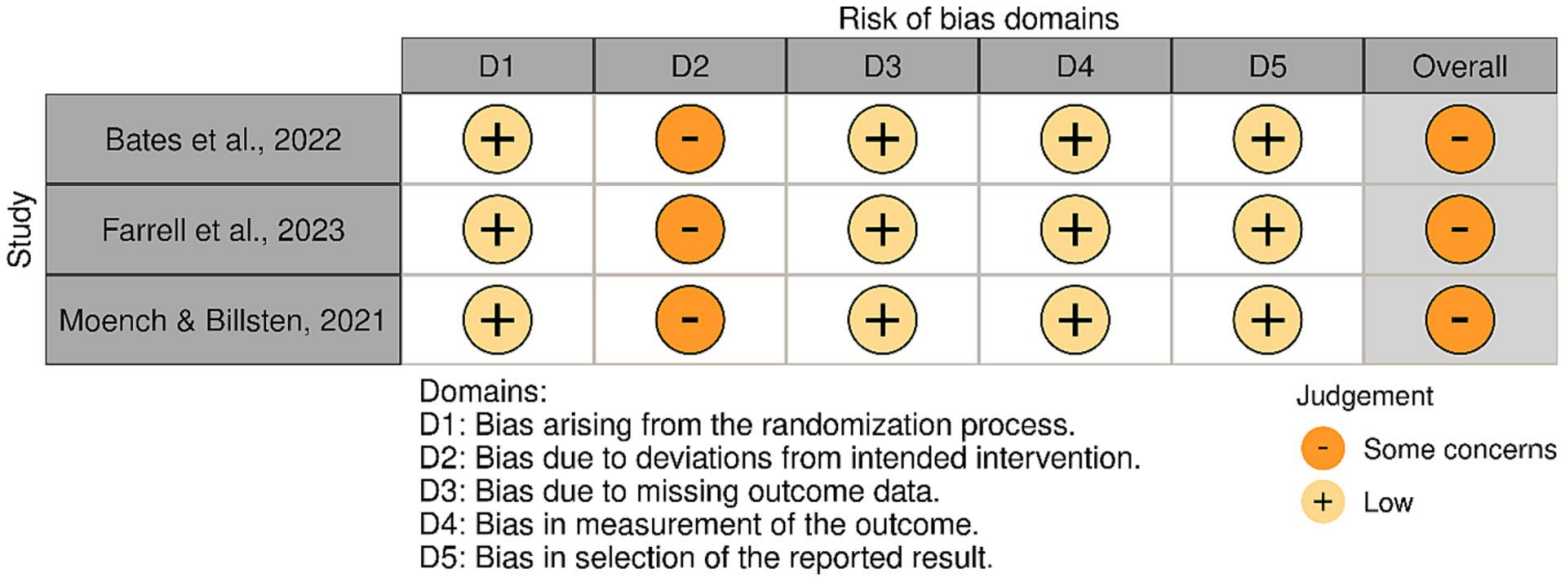
Quality evaluation of randomised studies.

The included articles varied regarding risk of bias. Figure 2 shows the assessment of the non-randomised studies. All studies received a moderate risk of bias. The most common shortcomings were the lack of blind assessors and the use of self-reported outcomes. Figure 3, shows the risk assessments of the randomised studies. Three randomised studies had some concerns of bias. This was due to allowing deviation from the intended intervention or the use of self-report measures only.

### 3.3 Findings

#### 3.3.1 Therapist delivered live online sessions

##### 3.3.1.1 Individual sessions

The Standard EMDR Protocol was assessed in two separate studies. The first study recruited EMDR therapists practicing in the UK and Ireland (30). A total of 33 therapists provided data on 93 different clients. The results demonstrated reductions in mean scores for the IES(R), GAD-7, PHQ-9, and PCL-5 checklists, indicating improvement in mental health difficulties. Yet, no significant correlation was found between the duration of EMDR training and clinical outcomes, nor was there a noteworthy difference in the connection between accreditation level and clinical outcome.

In a similar study conducted in Germany (31), data from 23 therapists who conducted 102 EMDR sessions with 76 patients were analyzed. The findings highlighted the potential efficacy and viability of online EMDR. The reduction in SUD was on the same level as that observed in face-to-face EMDR studies. Notably, the bilateral stimulation as eye movements led to greater reductions than tapping in sessions.

A separate study recruited a total of 24 participants for a single-session EMDR protocol known as the “Blind 2 Therapist” (VB2Tr), with measurements taken at pre-treatment, post-treatment, 1 month, and 6 month follow-ups (32). All 24 research participants completed the VB2Tr treatment session and subsequent measurements without any dropouts. The study demonstrated a substantial decrease in both SUD and emotional intensity.

The concluding study explored the effects of a single session of the Urgent EMDR (URG-EMDR) protocol on 17 healthcare professionals (33). The assessment focused on anxiety and depressive symptoms using the HAD scale, along with the level of perceived disturbance (SUD). The URG-EMDR protocol bears similarities to the standard EMDR protocol but aims to provide an intervention within 24 to 72 h following a critical incident. Seventeen female healthcare workers were involved in the study. The results indicated a significant difference between pre-test and post-test scores across all variables.

The R-TEP protocol was investigated across a total of six studies as an individual intervention, comprising 5 one-arm trials and one two-arm trial. The primary focus of these studies was to assess the impact of EMDR therapy on diverse mental health outcomes in participants, including health care professionals and patients.

Another study recruited healthcare workers diagnosed with post-traumatic stress disorder and utilized Beck Anxiety Inventory (BAI), Impact of Event Scale-Revised (EIS-R) and Maslach Burnout Inventory (MBI) (34). Each participant received five 90 min sessions. The study revealed significant decreases in mental health difficulties, with notable reductions in anxiety, depression, and emotional exhaustion symptoms that persisted at the 1 month follow-up.

In a separate study, out-of-home care staff who were regularly exposed to workplace-related trauma were recruited (35). Over a 3 years pilot study, both the Recent Traumatic Episode Protocol (R-TEP) and the Group Traumatic Episode Protocol (G-TEP) were administered. Due to the COVID-19 pandemic, individual EMDR (R-TEP) was provided online in 2020. The results indicated a substantial reduction in PCL-5 scores from baseline to follow-up, and participants who underwent R-TEP or G-TEP experienced symptom reductions. Both face-to-face and online deliveries exhibited significant reductions in PCL-5 scores, with no significant differences between the two modes of delivery.

Yurtsever and colleagues conducted another study employing online R-TEP with 154 individuals working alongside frontline professionals and COVID-19 patients (36). Participants underwent five sessions, and the analyses demonstrated the effectiveness of EMDR therapy in reducing PTSD levels across all groups. While PTSD levels in frontline professionals continued to decrease during the follow-up, they remained consistent in other groups.

In a different approach, Perri and colleagues recruited 38 patients diagnosed with acute stress disorder and randomly assigned them to receive either EMDR R-TEP or CBT treatment (37). Both groups underwent a 7-session therapy, resulting in significant reductions in anxiety, stress, and depression for both treatments.

Bates and colleagues conducted the sole RCT of R-TEP, enrolling 26 participants and randomizing them into online EMDR R-TEP or usual care groups (38). Participants were offered up to eight EMDR sessions until disturbance points were addressed. While an 83% intervention adherence rate was observed, and most participants completed all study procedures, no significant changes in anxiety or depression were noted. In a notable case study, Clarke explored the effects of nine sessions of online R-TEP on an intensive care survivor, indicating promising reductions in all outcome measures (39).

##### 3.3.1.2 Group sessions

Four studies examined the feasibility of administering various EMDR protocols as group interventions through online delivery.

In an RCT (40), frontline mental health and emergency workers were recruited to evaluate the effectiveness of EMDR G-TEP for treating Post-Traumatic Stress Disorder (PTSD) and moral injury. Participants were randomised into either a treatment group or a delayed treatment group. All participants underwent four online sessions of G-TEP. Measurements encompassed the International Trauma Questionnaire (ITQ), Generalised Anxiety Disorder 7-item (GAD-7), Patient Health Questionnaire (PHQ-9), Moral Injury Events Scale (MIES), and the EuroQol (EQ-5D) for quality of life. The results demonstrated a significant treatment effect in both the active and control groups.

IGTP was assessed as an online treatment option in two separate studies involving healthcare workers during the COVID-19 pandemic in Italy, conducted by the same research team. In the first study, 11 healthcare workers from a nursing home were recruited (41). Participants engaged in three EMDR group therapy sessions. The outcomes revealed a noteworthy reduction in post-traumatic stress disorder (PTSD) symptoms measured by IES-R, and there was a substantial enhancement in the quality of emotional experience following the intervention. However, when the IES-R questionnaire was administered to participants again at the 9 months follow-up, it indicated an increase across all subscales. In the same team’s second study, 587 healthcare workers were recruited (42). The IGTP protocol was delivered online, utilizing the same outcome assessment tools. Groups consisted of 4–6 participants, and the treatment comprised three meetings of approximately 2 h each over a month, occurring approximately once a week. All variables showed significant improvement after EMDR therapy, underscoring a clear treatment effect.

Lazzaroni and colleagues conducted a study involving 50 adolescents and young adults aged 13 to 24 years (43). This study diverged from prior R-TEP research by delivering R-TEP through three group sessions, each lasting 1 h. Pre- and post-treatment evaluations incorporated the Impact of Event Scale-Revised (IES-R), State–Trait Anxiety Inventory (STAI) scales, and the Emotion Thermometer. Additionally, the Post-Traumatic Growth Inventory (PTGI) questionnaire was administered post-intervention. The outcomes exhibited substantial improvements in STAI, IES-R, and Emotion Thermometer scores, indicative of reduced post-traumatic symptoms. Furthermore, a significant positive change was observed in PTGI scores.

#### 3.3.2 Self-administered computerised sessions

In addition to above studies, further attempts have already been made to extend the availability and accessibility of EMDR. Two studies have evaluated self-administered EMDR sessions of which one was a randomised controlled trial (44, 45).

In the first study standard EMDR protocol was again utilized using an artificial intelligence engine chatbot to fully replace clinicians (44). Moreover, the tool employed artificial intelligence (AI) to adjust the bilateral stimulation automatically based on the patient’s degree of responsiveness. A total of 31 participants participated in the single-session intervention, which incorporated various types of Bilateral Stimulation (BLS). The intervention consisted of four distinct phases. The initial three phases were formulated to diminish the intensity of emotions and beliefs linked to the traumatic event. The fourth phase was introduced to establish a positive belief concerning the same event. The IES-R and STAI instruments were administered as part of the pre-test assessment. Following this, four participants were excluded from the study based on their scores on the IES-R and STAI. These individuals were subsequently referred to a more specialized professional treatment. Following the single session intervention, participants showed the potential effectiveness of self-administered EMDR with the help of the AI tool in decreasing PTSD and anxiety.

In the second study on self-administered EMDR, Moench and Billsten conducted research during the COVID-19 pandemic involving healthcare workers and mental health clinicians (45). They implemented the EMDR-based protocol named Self-Care Traumatic Episode Protocol (STEP), which involved a single 90 mins session. This protocol, derived from the EMDR group treatment G-TEP, aimed to address trauma. The study encompassed a series of steps, starting with telephone assessment and screening. Following this, a 90 mins computerized session was conducted, where a recorded therapist guided the client through the protocol. Clients who managed to reduce their Subjective Units of Disturbance (SUD) level to 7 or lower after engaging in the 4 Elements activity were granted access to the intervention. The study included thirty-four participants who were randomly assigned to either the treatment group or a waitlist control. Assessments were conducted using the Generalised Self-Efficacy Scale (GSE) and the Depression and Anxiety Stress Scale (DASS-21). The results indicated a significant decrease in depression, anxiety, and stress levels, accompanied by an increase in general self-efficacy among the participants who underwent the STEP intervention.

## 4 Discussion

This systematic review aims to describe the current evidence regarding online/remote applications of EMDR therapy in patients with mental health difficulties. Overall, the results indicate that delivering EMDR therapy via online tools is feasible and potentially effective. This approach presents a promising alternative to in-person delivery of intensive trauma-focused therapy.

The studies in this review encompass a wide array of populations, including adults, adolescents, and healthcare workers, demonstrating the applicability of EMDR across various age groups and professional backgrounds. Some studies specifically target healthcare workers, emphasizing the need to address the mental health challenges within this occupational group. The delivery formats also vary, with studies utilizing individual live sessions, group live sessions, and self-administered computerized sessions.

### 4.1 Implications and recommendations

The application of online EMDR therapy seems to entail both advantages and disadvantages (32). On the advantageous side, this approach offers an alternative tool for reducing trauma symptoms, making it valuable in professional-limited environments. Furthermore, EMDR can be cost effective thanks to fewer sessions needed compared to CBT (46). Notably, in four studies in this review, the treatment was completed within a single session, suggesting that online EMDR could potentially serve as a time-efficient and cost-effective solution, especially in situations where there is a need for immediate intervention after incidents like work-related difficulties (34, 40, 44, 45).

Moreover, online EMDR can offer flexibility, adaptability, decrease in costs thanks to reduction in commute costs for both the clients and the therapists, and linguistic and cultural versatility, particularly for participants from already stigmatized groups (47) by conducing sessions from the privacy of one’s home which can help alleviate concerns about being seen entering or leaving a therapist’s office. By doing so, online EMDR might offer a more acceptable means of receiving therapy bypassing some cultural and social stigmas associated with seeking mental health help.

Furthermore, online EMDR’s capacity for intensive delivery and its integration into a comprehensive treatment package enhances accessibility. Although not included in this review, several articles are worth mentioning to showcase this. In these studies, EMDR has been successfully combined and integrated with other techniques (48–50). In the first study of its kind, EMDR therapy was administered utilizing a website (50) and combined with psychoeducation sessions. Fifteen adult patients diagnosed with PTSD were included and the findings revealed significant reductions in clinician-rated PTSD severity. However, there was no statistically significant decline in self-rated PTSD symptoms. In fact, three participants reported a worsening of self-rated PTSD symptoms after completing the treatment. In the second study, EMDR was offered as part of a comprehensive treatment program involving prolonged exposure, physical activities, and psychoeducation (48). This treatment was administered through telehealth over a 4 days period and the research involved six patients experiencing Complex PTSD. The findings revealed that four out of the six patients no longer met the diagnostic criteria for PTSD or Complex PTSD. In a subsequent study conducted by the same research team, EMDR therapy was once again combined with prolonged exposure, physical activities, and psychoeducation to treat 73 patients diagnosed with PTSD (50). The findings indicated that 60 patients (82.2%) no longer met the diagnostic criteria for PTSD, and the proportion of patients with Complex PTSD decreased from 47.1 to 10.1%.

On the other hand, this method relies heavily on technology and software access, which may not be universally available. Privacy and confidentiality issues may arise in the online context, and access to smart devices may become a prerequisite, limiting its applicability in certain areas for certain groups such as refugees and people with low finances. It may also not be well-suited for addressing severe mental health issues. Furthermore, concerns regarding potential risks accompany remote treatment, and the partial absence of non-verbal cues may also limit the therapeutic experience. Moreover, whether conducted in-person or online, the effectiveness of EMDR or any therapy depends on various factors, including the individual’s specific needs and the skills of the therapist. The low drop-out rates across studies indicate that participants generally tolerate online EMDR therapy well.

Processing experiences and memories within the context of EMDR treatments can sometimes lead to varying degrees of emotional distress and dysregulation (50). The capacity of clinicians to effectively address and manage these reactions might be constrained when conducting EMDR therapy online, potentially raising safety concerns that are challenging to navigate from a remote setting. The impact of EMDR therapists’ experience on treatment outcomes has been a subject of debate. Some studies indicate that EMDR therapy shows improved results with sessions lasting longer than 60 min (51) and when administered by more experienced therapists (18). However, this review did not find a direct connection between accreditation level and clinical outcomes (30). Additionally, the reduction in SUD was not linked to the age or gender of both therapists and patients (31).

As another challenge, the technological challenges can hinder the delivery of sessions and introduce distractions that may compromise the overall effectiveness of EMDR treatment (25, 26). A lack of focus, fatigue, or exhaustion stemming from online communication and difficulties related to the application of bilateral stimulation due to technical challenges can also be important difficulties during online EMDR sessions (52).

Future research should (a) evaluate the cost-effectiveness of online EMDR in comparison to other online interventions; (b) identify moderators and mediators that might influence treatment outcomes, such as the number of sessions, gender, age, facilitator characteristics, and the number and characteristics of traumas experienced and (c) incorporate qualitative techniques involving both participants and facilitators to understand the reasons for dropouts and withdrawals to develop strategies to improve recruitment. Finally, the majority of studies recruited adults, with some including adolescents. Although several systematic reviews indicated the effectiveness of EMDR for child and adolescent populations for a range of mental health conditions (20, 46, 53–58), the lack of studies targeting children in the context of online EMDR indicates a potential gap in the existing research. This suggests the need for more focused and comprehensive investigations into the efficacy and application of online EMDR specifically in children and adolescents.

### 4.2 Limitations

While this review presents promising findings, it is important to note that some studies within it exhibit significant limitations despite the importance of the quality of methodology in EMDR trials (59). These limitations include the lack of a control group, small sample sizes, absence of follow-up assessments, and reliance solely on self-report measures when evaluating the quality of evidence. Similarly, fidelity assessment was reported in only three out of the sixteen studies (32, 36, 40). In these studies, fidelity assessment was carried out through either recording of the sessions or group supervision.

Several systematic reviews indicate that, EMDR therapy has been mostly shown effective or promising for various other populations when offered face to face, including dementia patients with PTSD (60), incarcerated people with PTSD (61), and refugee and asylum seeker populations with PTSD (62, 63). Furthermore, other systematic reviews showed similar results for in person EMDR for various other conditions including but not limited to psychosis symptoms (64, 65), chronic pain (66, 67), functional neurological disorder (68), and bipolar affective disorder (69). However, even though this review included several populations (adults, adolescents, and healthcare professionals) for PTSD, depressive and anxiety symptoms, this review is unable to comment on the effectiveness of remote EMDR for above populations and conditions due to lack of research. Finally, the study’s protocol was registered retrospectively on INPLASY. A retrospective registration may be seen as a limitation; however, despite the lack of the initial registration, no substantial change was made on the protocol since its inception. Furthermore, to ensure transparency and good quality, multiple reviewers were involved in the screening and selection of the articles as detailed in the Methods section.

## 5 Conclusion

In summary, despite its limitations, the trials included in this review indicate that online EMDR shows promise as a valuable tool in alleviating PTSD symptoms and addressing other mental health difficulties. However, due to methodological challenges, there is a clear need for studies with more robust designs, larger sample sizes, validated assessment tools, and follow-up evaluations. Future comparative trials could shed light on how different delivery methods impact treatment outcomes, offering insights into the significance of group interaction in treatment effectiveness beyond just the treatment content itself.

## Data availability statement

The original contributions presented in the study are included in the article/supplementary material, further inquiries can be directed to the corresponding author.

## Author contributions

SK: Conceptualization, Methodology, Writing – original draft, Writing – review & editing. ZK: Methodology, Writing – review & editing, Conceptualization, Investigation, Writing – original draft. AA: Investigation, Conceptualization, Writing – review & editing, Writing – original draft, Methodology.
